# 7 Tesla MRI Followed by Histological 3D Reconstructions in Whole-Brain Specimens

**DOI:** 10.3389/fnana.2020.536838

**Published:** 2020-10-06

**Authors:** Anneke Alkemade, Kerrin Pine, Evgeniya Kirilina, Max C. Keuken, Martijn J. Mulder, Rawien Balesar, Josephine M. Groot, Ronald L. A. W. Bleys, Robert Trampel, Nikolaus Weiskopf, Andreas Herrler, Harald E. Möller, Pierre-Louis Bazin, Birte U. Forstmann

**Affiliations:** ^1^Integrative Model-Based Neuroscience Research Unit, University of Amsterdam, Amsterdam, Netherlands; ^2^Department of Neurophysics, Max Planck Institute for Human Cognitive and Brain Sciences, Leipzig, Germany; ^3^Neurocomputation and Neuroimaging Unit, Department of Psychology and Educational Science, Free University Berlin, Berlin, Germany; ^4^Department of Experimental Psychology, Utrecht University, Utrecht, Netherlands; ^5^The Netherlands Institute for Neuroscience, Institute of the Royal Netherlands Academy of Arts and Sciences, Amsterdam, Netherlands; ^6^Department of Anatomy, University Medical Center Utrecht, Utrecht University, Utrecht, Netherlands; ^7^Department of Anatomy and Embryology, Maastricht University, Maastricht, Netherlands; ^8^NMR Methods & Development Group, Max Planck Institute for Human Cognitive and Brain Sciences, Leipzig, Germany; ^9^Department of Neurology, Max Planck Institute for Human Cognitive and Brain Sciences, Leipzig, Germany

**Keywords:** *post mortem* human brain, ultra-high field MRI, whole brain imaging, histology, formalin fixation

## Abstract

*Post mortem* magnetic resonance imaging (MRI) studies on the human brain are of great interest for the validation of *in vivo* MRI. It facilitates a link between functional and anatomical information available from MRI *in vivo* and neuroanatomical knowledge available from histology/immunocytochemistry. However, linking *in vivo* and *post mortem* MRI to microscopy techniques poses substantial challenges. Fixation artifacts and tissue deformation of extracted brains, as well as co registration of 2D histology to 3D MRI volumes complicate direct comparison between modalities. Moreover, *post mortem* brain tissue does not have the same physical properties as *in vivo* tissue, and therefore MRI approaches need to be adjusted accordingly. Here, we present a pipeline in which whole-brain human *post mortem in situ* MRI is combined with subsequent tissue processing of the whole human brain, providing a 3-dimensional reconstruction via blockface imaging. To this end, we adapted tissue processing procedures to allow both *post mortem* MRI and subsequent histological and immunocytochemical processing. For MRI, tissue was packed in a susceptibility matched solution, tailored to fit the dimensions of the MRI coil. Additionally, MRI sequence parameters were adjusted to accommodate T1 and T2^∗^ shortening, and scan time was extended, thereby benefiting the signal-to-noise-ratio that can be achieved using extensive averaging without motion artifacts. After MRI, the brain was extracted from the skull and subsequently cut while performing optimized blockface imaging, thereby allowing three-dimensional reconstructions. Tissues were processed for Nissl and silver staining, and co-registered with the blockface images. The combination of these techniques allows direct comparisons across modalities.

## Introduction

Magnetic resonance imaging (MRI) is today’s leading technique in the study of the human brain (Bandettini, 2009). Visualization of small brain areas in MRI data is challenging in view of the limited anatomical specificity that can be obtained using *in vivo* structural MRI scans. *In vivo* neuroimaging techniques are constantly moving forward, but still do not allow the visualization of all individual brain structures (Turner, 2016). Particularly small structures located in the human subcortex continue to provide challenges (Alkemade et al., 2013; Forstmann et al., 2017). Similarly, the robust visualization of individual neocortical layers, which each are 200–300 μm thick, is currently limited to certain cortical areas, e.g., the primary visual cortex, but fails for whole-brain approaches (Trampel et al., 2019). This can, in part, be attributed to motion artifacts of various causes, including pulsations (Weiskopf et al., 2015; Godenschweger et al., 2016), which do not play a role in research in *post mortem* MRI. Scanning *post mortem* human brain specimens at ultra-high fields (UHF) can provide a level of detail and precision that is not achieved using any non-invasive *in vivo* neuroimaging approach. Unlike studies in living subjects, the study of *post mortem* human brain anatomy allows for extensive scan periods (e.g., hours or days). As a consequence, many MR sequence repetitions can be acquired and aggregated, thereby substantially improving the signal-to-noise ratio (SNR). The resulting *post mortem* scans with their unmatched detail can be used to assist anatomical orientation *in vivo* through the creation of detailed UHF-MRI atlases (Forstmann et al., 2017). Additionally, *post mortem* imaging studies have the potential to bridge the gap between classic histological studies, immunocytochemical approaches, *in situ* hybridization, and *in vivo* UHF-MRI studies (Forstmann et al., 2017).

Preparing *post mortem* tissue for UHF-MRI, however, poses its own challenges. Tissue fixation is commonly performed in formaldehyde, and archival specimens are often stored in formaldehyde solutions for extended periods of time (years). Uneven fixation of the tissue will result in MRI artifacts (Yong-Hing et al., 2005), and storage in formaldehyde for more than 6 years can result in the formation of formaldehyde crystals, which interfere with MRI acquisitions (van Duijn et al., 2011). An additional challenge when aiming to bridge the gap between histological and/or immunocytochemical techniques and MRI research is the preparation of the tissue so that it accommodates both optimal MRI acquisition as well as it preserves the tissue properties required for reliable analyses using microscopy approaches. Tissue processing for microscopy purposes, causes substantial tissue deformation, caused by brain extraction, as well as the preparation of slides for histology and/or immunocytochemistry. These deformations need to be accommodated in co-registrations of *post mortem* and *in vivo* MRI, as well as MRI to histology.

**FIGURE 1:**
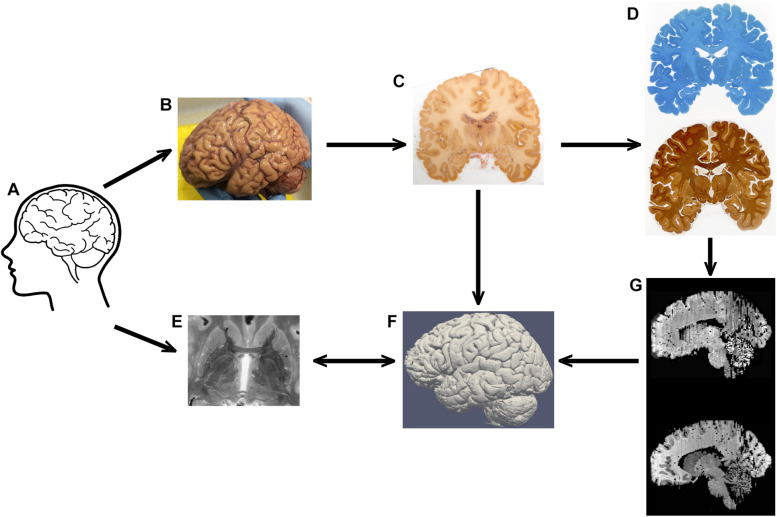
Representation of the tissue processing. Tissue **(A)** is subjected to MRI scanning, after which autopsy is performed (note that the brain is still covered by the meninges in his image) **(B)**. After blockface imaging **(C)**, sections are stained for Nissl or silver **(D)**. MRI contrast **(E)** and blockface reconstructions **(F)**, and section reconstructions **(G)** are aligned in the same space.

For state-of-the-art UHF-MRI of *post mortem* tissue specimens, custom designed packaging systems and, in a subset of cases, specialized MRI hardware is used (Seehaus et al., 2013; Weiss et al., 2015). Here, we present a protocol (Figure 1) that allows ultra-high resolution MRI on human *post mortem* whole brain specimens using basic materials. We use standard commercial hardware for *post mortem* MRI scanning. We performed scanning of the brain inside the skull, thereby preventing tissue deformation and damage, and facilitating co-registration of the data with *in vivo* whole-brain scans. Furthermore, we show that blockface images allow a three-dimensional (3D) reconstruction of the brain volume. The obtained individual sections can be used for histological processing to perform anatomical validation of MRI results visualizing small anatomical structures which can then be reconstructed providing a detailed account of the 3D microstructure of the human brain. We would like to acknowledge that the technical execution of the protocol requires an extensive set of skills and a range of equipment which is unlikely to be available within a single research group. Implementation of the protocol therefore relies on an interdisciplinary collaboration of the fields of anatomy, neuroimaging and microscopy. The protocol description presented here can be used as a guide to set up the required collaborations.

## Methods and Results

### Tissue and Fixation Procedure

Specimens stored in a formaldehyde solution were obtained through the whole-body donation program at the University of Maastricht (Table 1). Donors did not have any record of clinical neurodegenerative or neurological disease. All donors provided written informed consent for whole body donation prior to death, and tissue was obtained in accordance with the Dutch Burial and Cremation act. Additional approval for these studies was obtained from the Ethical Board of the Medical Faculty of the University of Leipzig (153/17-ek). Tissue was routinely fixed within approximately 24 h after death using perfusion fixation through the femoral artery with a 2.3% formaldehyde concentration with 20% ethanol (V/V) for perfusion, followed by 2.3% formaldehyde concentration with 10% ethanol (V/V) for post fixation for 30 days. A small amount of methanol is added to prevent spontaneous condensation polymerization. Ethanol is used for fixation and permeabilization of fatty substances without solubilizing them, 8.3% glycerol (V/V) is added to reduce stiffness of muscle tissue. Tissue fixation is therefore based on an initial alcohol fixation, which is followed by a cross-linking reaction (Fox et al., 1985). Bodies were perfused at 4 atmosphere for 2.5 h using a total volume of 8–15 L formalin depending on body and perfusion circumstances at a speed of 1–2 L per hour, followed by immersion post fixation for 30 days. Tissues were stored in formaldehyde until further processing. In preparation for scanning, tissue was rinsed for 45 min in running tap water to remove the formaldehyde solution, and then transferred to phosphate buffered saline (PBS, pH 7.4) with 0.05% sodium azide [(pH ∼ 6.6–7.0): 145 mM NaCl, 9 mM disodium phosphate (Na_2_HPO_4_ cat. no. 71640, Sigma-Aldrich, St. Louis, MO, United States), 2 mM sodium phosphate (NaH_2_PO_4_.H_2_O, cat. no. S9638, Sigma-Aldrich, St. Louis, MO, United States)] 0.05% sodium azide (cat.no. S-2002, Sigma-Aldrich, St. Louis, MO, United States) for at least a month.

**TABLE 1:** Post mortem specimens.

Specimen #	Autopsy number	Age (y)	Sex (M/F)	Formaldehyde storage time (y)	Remarks
1	32/2014	79	M	3	MRI acquired, used for histological processing, parietal lesions
2	14/2016	61	F	1	MRI acquired
3	20/2016	70	F	3	MRI acquired
4	13/2017	82	F	2	MRI acquired
5	04/2017	91	F	2	MRI acquired
6	12/2017	59	F	2	MRI acquired, used for histological processing
7	05/2017	80	M	1	MRI acquired, used for histological processing
8	15/2017	75	F	1	MRI acquired, used for histological processing
9	12/2015	64	F	3	No MRI acquired, used for histological processing

*F, female; M, male; y, years.*

### Packing Procedures

We tested for a potential beneficial effect of trepanation by drilling two holes on the sagittal suture (4 mm diameter) to allow air caught in the skull to escape. The effects of trepanation, and submerging the specimen in PBS, in combination with creating a PBS flow resulted in the removal of a part of the air trapped in the skull, as evidenced by escaping air bubbles. Specimens were transferred to a heavy duty waterproof rubble bag (cat. no. 079390, Gamma, Leusden, Netherlands). Heads were positioned so that the severed neck faced the opening of the bag. The bag was shaped tightly around the specimens using duct tape Tesa Extra Power Tape 50 m × 50 mm (Gamma, Amsterdam, Netherlands), and submerged in a bucket of water to remove the air on the outside of the specimens through buoyancy. The specimens were vigorously agitated, while being submerged to remove air. The bag was compartmentalized using food clips (Bevara, Ikea) to create communicating vessels to allow remaining air to escape further. The specimens were placed in a second bag providing extra protection against leakage. As result of this embedding and packing procedures tissue was surrounded by susceptibility matched solution, which also filled nasal, sinus and ear cavities mitigating susceptibility artifacts induce by air filled cavities in *in vivo* MRI scans.

### Ultra-High Field MRI Scanning

Magnetic resonance imaging was performed using a MAGNETOM 7T whole-body system (Siemens Healthineers, Erlangen, Germany) with a circularly polarized radio-frequency (RF) transmit/32-channel receive head array coil (NOVA Medical Inc., Wilmington, MA, United States). UHF-MRI parameters were determined based on prior experience and additional test scans including quantitative imaging, particularly T1 and T2^∗^ maps (Weiss et al., 2015). We included a 3D multi-echo spoiled gradient-recalled echo (GRE) sequence, since these scans allow for an easy identification of the remaining air inside the specimen by the shortened T2^∗^ due to susceptibility-induced local magnetic field gradients. To reduce the strain on the MRI gradient system (i.e., heating), we inserted pauses between those scan protocols which relied on high gradient duty cycles (a 10 min pause in-between every hour of continuous scanning). Adjustment of the RF frequency was performed at the start of each sequence to reduce frequency drift-related apparent sample shifts associated with prolonged high-duty-cycle scanning.

The structural MRI data for whole-head specimens was acquired using a multi-parametric mapping technique (Weiskopf et al., 2013) further adapted for 7T and for the substantially increased isotropic resolution of (0.40 mm)^3^. The protocol consisted of three 3D multi-echo GRE scans with T1, proton density (PD-) and magnetization-transfer (MT-) weightings, RF pulse flip angle (FA) = (38, 7, 7) degrees, number of echoes = (8, 8, 6), echo times (TE) equally spaced between 3.4 and 21.6 ms, repetition time (TR) = 31.8 ms. MT weighting was achieved by a 4 ms long Gaussian RF pulse (3 kHz off-resonance, peak amplitude 2.0 μT, BW = 450 Hz) applied once per TR. The sequence was both RF- and gradient-spoiled. With a matrix size of 480 × 640 × 416 (phase × read × partition) the acquisition time per contrast was 1 h and 46 min. The set of contrasts was repeated four times for obtaining sufficient SNR. Besides weighted images, maps of the RF magnetic field were acquired for B1 + correction (Lutti et al., 2012). The data of specimen #1 and #2 were acquired with an earlier version of the protocol with identical parameters except for threefold acceleration applied in the partition phase encoding direction and an increased number of repetitions (*n* = 12). After offline image reconstruction with SENSE, magnitude data from the multiple repetitions were averaged together before further processing. Quantitative maps of longitudinal relaxation rate (R1), proton density (PD), magnetization transfer (MT) and effective transversal relaxation time (R2^∗^) were then calculated with the hMRI toolbox^1^ within the SPM 12 framework^2^ and MATLAB. R2^∗^ maps were calculated by ordinary least squares fitting to the multi-contrast data (Weiskopf et al., 2014).

To assess the effects of brain extraction on the occurrence of air bubbles we performed two additional, identical scans at the Spinoza Centre for Neuroimaging in Amsterdam, Netherlands using a Philips Achieva 7T MRI scanner with a 32-channel head array coil. T1w, T2^∗^ contrasts were obtained using a MEMP2RAGE (multi-echo magnetization-prepared rapid gradient echo) sequence, as described previously (Caan et al., 2019). First the scan was performed in *in situ* specimens #7 and #8. The scan was then repeated after extraction of the brain after gentile mechanical agitation to try and remove the air from the specimens, before the removal of the meninges.

**FIGURE 2:**
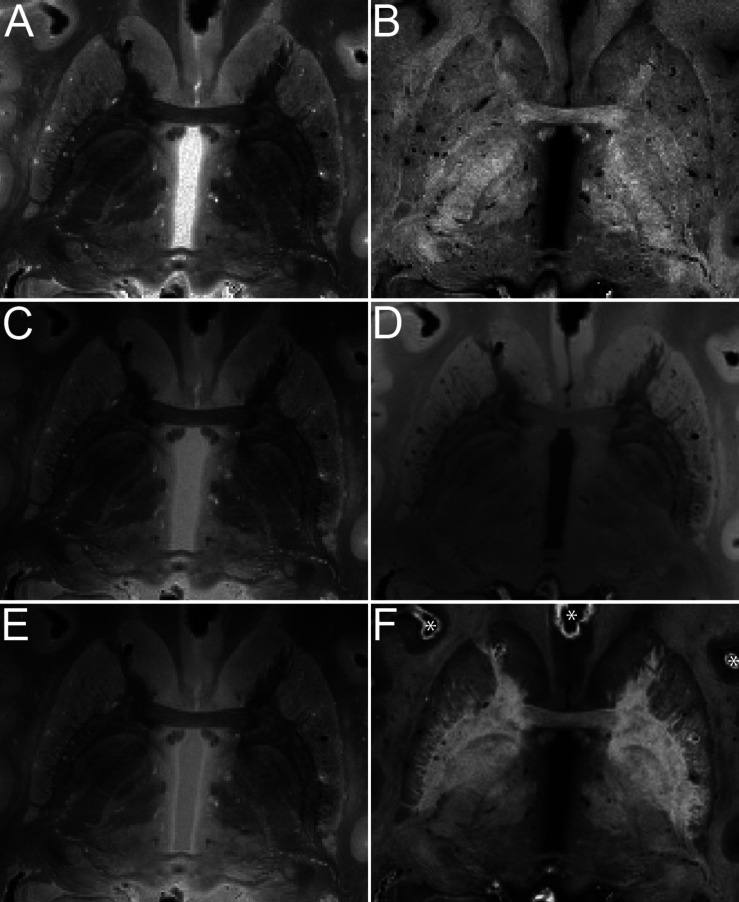
Quantitative maps of MR parameters and MRI images of the basal ganglia of specimen #8 (axial view). Contrasts are proton density **(A)**, longitudinal relaxation rate R_1_ **(B)**, MTw **(C)**, T_1_w **(D)**, PDw **(E)**, and OLS R${}_{2}^{*}$ **(F)**. Asterisks in F indicate air bubbles.

MRI images of the brains were, as expected, very sharp, as a result of the averaging over scan repetitions, as well as the absence of movement artifacts (Figure 2). No artifacts resulting from uneven fixation were observed.

We found that air was introduced to all specimens. We therefore investigated whether brain extraction combined with mechanic agitation would help the air to escape by performing a direct comparison before and after brain extraction, which resulted in a shift, rather than the disappearance of the air. This was observed on the MRI contrasts as regions of hyperintensities as well as dark bands occurred in the vicinity of air bubbles as a result of the induced magnetic field gradients. The consecutively acquired scans can be overlaid directly without the need for registration since no movement occurs, although caution should be used in light of possible scanner drifts. Distortions resulting from tissue-air interfaces at the level of the oral and nasal cavities were not observed, since these cavities were filled with susceptibility matched PBS.

### Tissue Cutting for Histology and Immunohistochemistry

We prepared five specimens for histological processing (#1, 6–9). After brain extraction and removal of the meninges, tissues were saturated in sucrose to prevent freezing damage using increasing volumes of sucrose (1 week 15%, 1 week 20%, 1 week 25%, 3 × 2 weeks 30%). Formalin fixation is known to cause slight tissue swelling (Tran et al., 2017). We did not determine brain weight, since determination of fixed tissue weight does not provide any biologically relevant information. We processed these brains further as proof of concept to demonstrate that our entire protocol can be executed reusing the same tissue samples. After saturating the tissue with sucrose, the brains were slowly frozen on dry ice, and embedded in TissueTek OCT compound (Sakura Finetek United States). This compound provides a high contrast between the brain tissue and the surrounding medium in blockface images, which enabled efficient 3D reconstruction. The tissue was mounted in a cryomacrotome (CM3600 XP, Leica Biosystems, Nussloch, Germany) and cut in 200 μm thick serial coronal sections. The field of travel of the knife was smaller than the length of the rostrocaudal brain axis, and therefore the knife had to be manually adjusted once during the cutting of each brain. This resulted in two sections of unknown thickness at this level, which were discarded. Blockface images were acquired at these levels, and all other sections were collected for further processing. The cryomacrotome was housed in a convection-cooled large-volume cryochamber. The low temperatures were achieved and maintained by cold air circulating inside the cryochamber, and a fan controlled the air circulation speed. The temperature was monitored digitally throughout the cutting process to ensure a stable temperature environment. Prior to each sectioning stroke, a stepper motor fed the microtome knife downwards toward the specimen 200 μm controlling section thickness.

The cryomacrotome temperature was kept stable at −16°C. For specimen #1, and 6–8 the blockface of the cutting plane was photographed for each individual slice (Leica DFC450 C, Leica Microsystems, Nussloch, Germany), and these images were used to create a 3D surface reconstruction. Individual sections were stored in separate 60 ml containers (VWR Catalog No: 216-2621) in a cryoprotectant solution (30% glycerol, 30% ethylene glycol, 40% 1 × PBS) and stored at −20°C until further processing.

**FIGURE 3:**
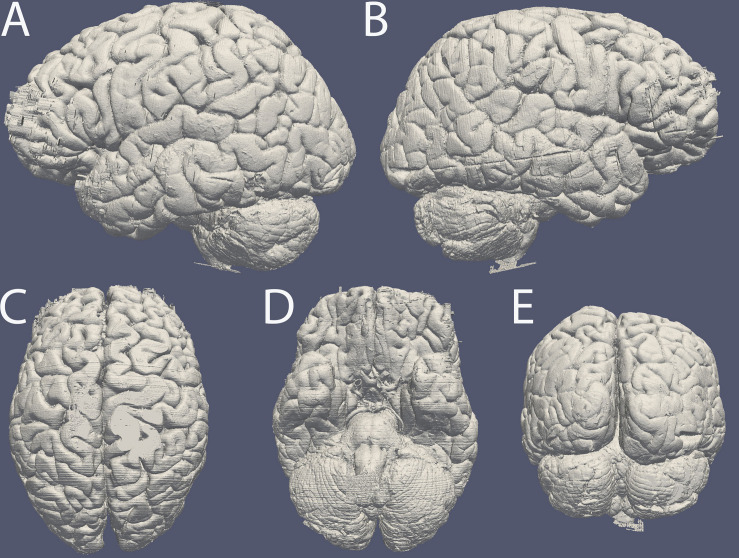
Surface reconstruction of specimen #8 based on blockface images created in MIPAV. Surface of the **(A)** left and **(B)** right hemisphere, **(C)** dorsal surface, **(D)** ventral surface, **(E)** occipital lobe and cerebellum. Note the artifacts caused by incomplete coverage in OCT in the frontal pole. The line along the side of the entire right hemisphere is the reconstruction of a cut resulting from the autopsy procedure.

After brain autopsy specimens #1, and 6–8 were sucrose protected and we performed cutting in combination with blockface imaging. Images were stacked in ImageJ without requiring any registration steps (10.21942/uva.12496853.v1). Based on these image stacks a 3D reconstruction was created (Figure 3).

### Tissue Staining

For mounting sections were thawed and rinsed extensively in Tris-buffered Saline (TBS): 150 mM NaCl (cat. no. 1.06404.1000, Merck, Darmstadt, Germany), 50 mM Tris–HCl, pH 7.6 (Trizma cat. no. T1503, Sigma-Aldrich, St. Louis, MO, United States)] to remove sucrose and the cryoprotectant solution. Sections were dried at 37^o^C for several days. Sections were delipidated through xylene (2 × 45 min), followed by rehydration through a graded ethanol series. For Nissl staining, sections were rinsed in distilled water (3 × 5 min), and dipped in a 0.17% thionin, glacial acetic acid was added to the solution to improve differentiation. After rinsing in running tap water, sections were dehydrated in a graded ethanol series, cleared in xylene and coverslipped using entellan. Nissl stains were used for anatomical orientation, and sections containing insular, temporal, frontal, parietal, occipital and cerebellar cortex, cingulum, globus pallidus and caudate and putamen, hippocampus, substantia nigra, subthalamic nucleus, red nucleus, dentate nucleus, the locus coeruleus, pontine base, and olivary nucleus were stained for hematoxylin and eosin for neuropathological assessment. To assess staining quality, we routinely checked the penetration depth of the staining by placing sections under the microscope inspecting the entire depth of the section at several locations in the section.

For Bielschowsky silver staining, we incubated the sections in 5% silvernitrate solution for 45 min at 40^o^C under dark conditions. Sections are rinsed in MilliQ water, and ammonium hydroxide was added to a fresh silver nitrate solution at 40^o^C until the precipitate is dissolved. Sections were incubated for 30–45 min at 40^o^C under dark conditions. We used approximately 0.1 ml of staining solution per cm^2^ for all staining solutions. One percent ammoniumhydroxide was added to the sections and incubated for 60 s. Sections were rinsed in MilliQ water. A fresh ammoniated silver nitrate solution was prepared to which developer was added. Sections were incubated in the developer solution for 40–45 min at room temperature (RT), and were kept afterward in MilliQ water. To reach optimal labeling of axons a second round of developing was performed by incubating slides for 45 min in freshly prepared ammoniated silvernitrate-developer mixture, during which the progress was monitored under the microscope. Sections were washed in MilliQ water for 5 min, followed by 5 min in 5% thiosulphate to remove the excess silver. After a short rinse in MilliQ water, sections were transferred to 50% ethanol. Sections were dehydrated through a graded ethanol series, cleared in xylene, and coverslipped in entellan.

For calbindin (CALB) and parvalbumin (PARV) immunohistochemistry sections were thawed and rinsed in TBS. For CALB and CALR we briefly equilibrated the sections in 50 mM Tris-HCl (pH 9). Tris-HCl was replaced and antigen-retrieval was performed by incubation in a waterbath at 90°C for 30 min. No antigen-retrieval was performed for PARV. At RT endogenous peroxidase was inactivated by incubation in TBS containing 3% H_2_O_2_ for 30 min at RT. After incubation in Supermix (SUMI: Tris-buffered Saline TBS containing 0.25% gelatin (cat. no. 1.04078.1000, Merck, Darmstadt, Germany) and 0.5% Triton X-100 (cat.no. X100, Sigma-Aldrich, St. Louis, MO, United States)] for 10 min antibodies were transferred to the primary antibody solution. Sections were incubated overnight at 4°C in either rabbit-anti-CALB (1:7,500 in SUMI, CB-38a, lot # 9.03, Swant), mouse-anti-CALR (1:7,000 in SUMI, 6B3, lot # 010399, Swant), or mouse-anti-PARV (1:8,000 in SUMI PV235, lot # 10-11 [F], Swant), after washing in TBS (pH 7.6 for 3 × 10 min at RT) sections were incubated in SUMI for 10 min in SUMI, after which sections were incubated in the appropriate biotinylated secondary antibody in SUMI for 1 h at RT. All incubations were performed under constant mechanical agitation. After washing in TBS (3 × 10 min at RT) and 10 min incubation in SUMI at RT sections were transferred to an avidin-biotinylated complex (ABC: VECTASTAIN ABC Kit: cat. no. PK-6100, Vector laboratories Inc., Burlingame, CA, United States), followed by washing in TBS and SUMI. To visualize immunoreactivity sections were incubated in SUMI containing 0.5 mg/ml 3,3′-diaminobenzidine (DAB: cat. no. D5637, Sigma-Aldrich, St. Louis, MO, United States) and 0.01% H_2_O_2_ under constant agitation. DAB reaction was stopped by dilution in SUMI instead of water, which prevented tissue clumping. Sections were then transferred to TBS-0.1%Triton X-100 and mounted onto gelatin-coated slides. Sections were dried overnight at 37°C, dehydrated through a series of graded ethanols, cleared in xylene and coverslipped using entellan. Sampling intervals were 1:6 for silver, PARV, CALB, and CALR staining and 1:3 for Nissl staining in the subcortex. In the remainder of the brain we stained consecutive sections for PARV, Nissl, and Silver, resulting in a 1:3 sampling interval for each stain.

**FIGURE 4:**
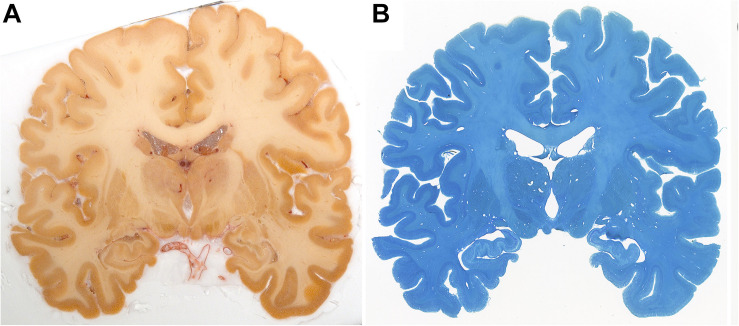
Blockface image of specimen #8 **(A)** and the corresponding Nissl **(B)** stained sections.

**FIGURE 5:**
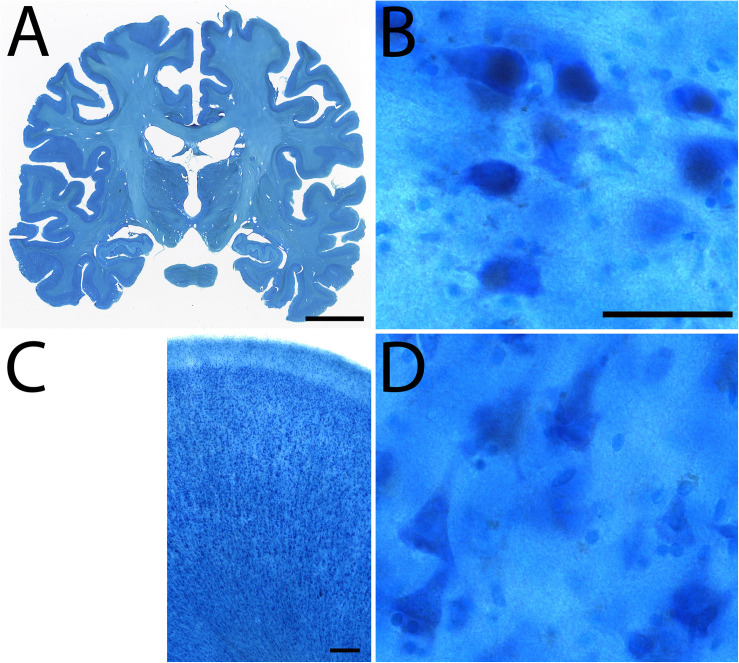
Examples of Nissl staining in specimen #8. Coronal section **(A)**, Subthalamic nucleus **(B)**, Cingulate cortex **(C)**, high power magnification of the cingulate cortex **(D)**. Scale bar represents 2 cm **(A)**, 50 μm **(B,D)**, 250 μm **(C)**.

**FIGURE 6:**
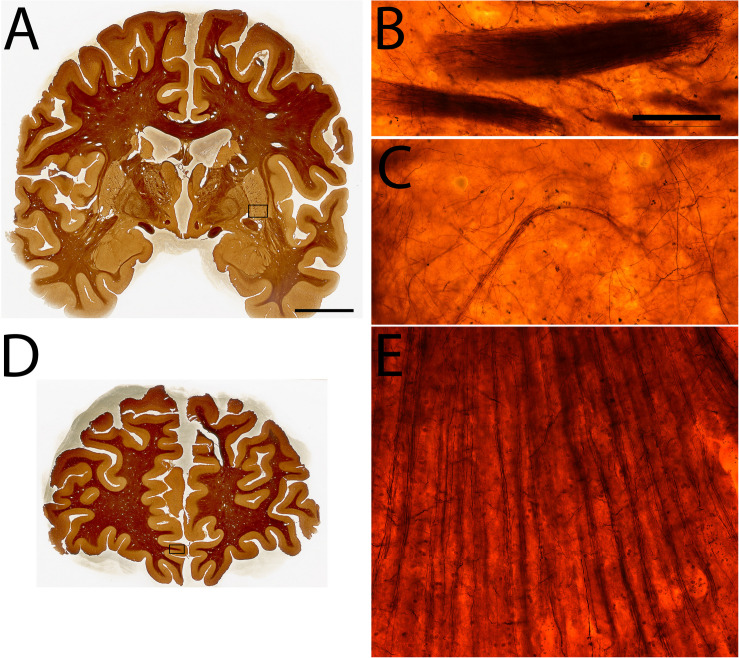
Examples of Silver staining in specimen #8. Coronal sections **(A,D)**, High power magnifications of the ventral putamen **(B,C)**, and the inferior rostral gyrus **(E)**. Scale bar represents 2 cm **(A,D)**, 50 μm **(B,C,E)**.

**FIGURE 7:**
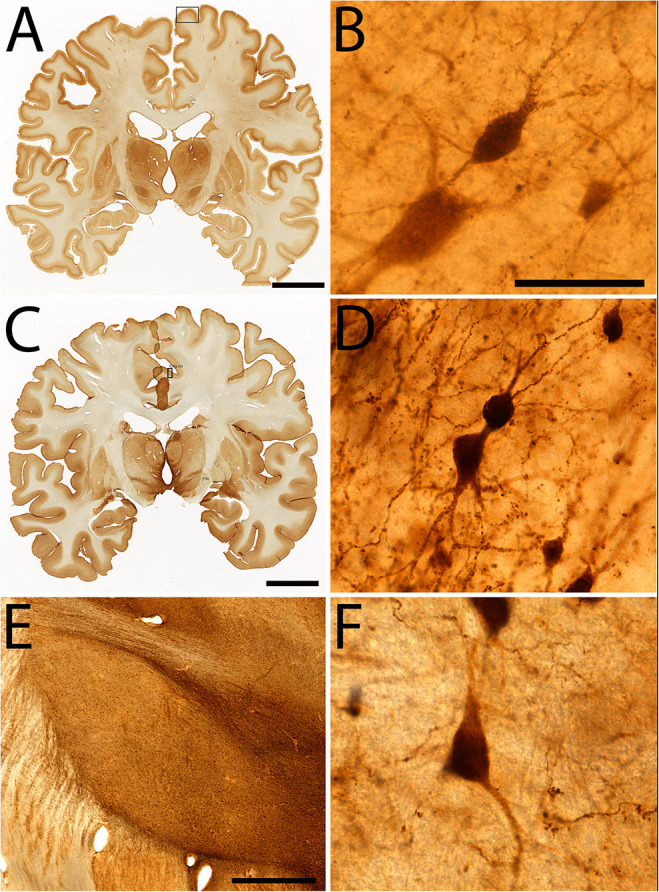
Examples of immunohistochemical staining in specimen #8. Coronal section stained for parvalbumin **(A)**, High power magnification of parvalbumin staining of layer III of the paracentral lobule **(B)**, Coronal section stained for Calretinin **(C)**, High power insert of layer III of the cingulate cortex **(D)**. Calretinin staining of the subthalamic nucleus **(E,F)**. Scale bar represents 2 cm **(A,C)**, 50 μm **(B,D,F)**, 200 μm **(E)**. Squares in **(A,C)** indicate the area in which **(B,D)** were sampled.

Sections were stored in cryoprotectant solution until further processing. Tissue was used for further histological processing. Substantial tissue deformations were observed and attributed to various sources, including cortical damage as a result of mechanical stress applied during the autopsy procedure, and widening of the sulci as a result of the removal of the meninges (Figure 3). Additionally, in subject #1 tissue integrity was negatively affected as a result of a lesion present in the parietal cortex of subject #1, and specimen #7 displayed major cryodamage in the caudal parts of the brain. Representative images of specimen #8 are displayed in Figures 4–7. Stained sections were digitized using an EPSON Perfection V700 Photo (Dual lens system) scanner set up for film scanning. Images were scanned using 1,200 pixels per inch.

Bielschowsky and Nissl stains that were obtained with a 1.2 mm slice interval (1:6 sampling interval) were co-registered with the corresponding blockface images, allowing the creations of 3D reconstructions of both the silver and Nissl stain in the same space. Thereby creating an integrated cytoarchitectural and axonal representation of the specimen.

### Image Processing

**FIGURE 8:**
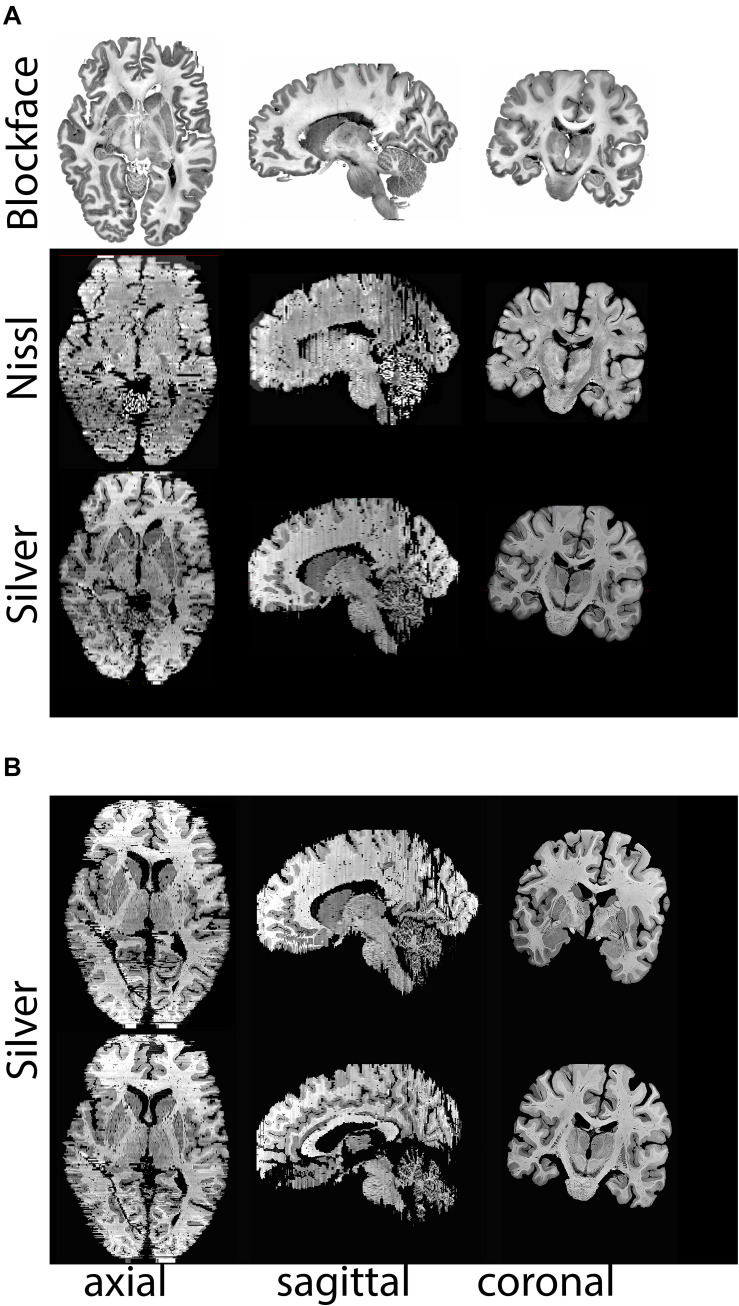
**(A)** Realignments/reconstructions of the blockface images of specimen #8 (top panel), Nissl stained sections (middle panels), and silver stained sections (lower panels), **(B)** Additional views of silver staining at different levels. Stained sections have a slice gap of 1.2 mm. Note that the coronal plane represents the cutting plane, axial and sagittal sections were realigned without any registration steps. Nissl and silver sections were registered to the corresponding blockface images. For registration purposes color images were transformed to gray scales of saturation, thereby inverting the appearance of the staining. Note that in areas in which small cortical pieces are detached as a result of cutting in the coronal plane, some tissue loss at the outer surface of the brain is observed.

**FIGURE 9:**
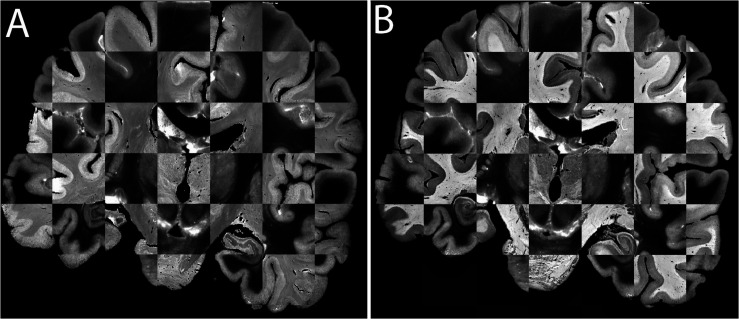
**(A)** Registration of a Nissl section (lighter contrast) to the blockface image (darker contrast). **(B)** Registration of a Silver stained section (lighter contrast) to the blockface image (darker contrast).

Blockface images were obtained using a camera setup with a built-in photostop in the macrotome. This ensures the exact same location of the tissue sample for each image, allowing to stack the images without any further realignment steps for further processing. The individual blockface images were stacked and transformed from color to a grayscale index of saturation, which separates well brain tissue from the background (Figure 8). Images with unusual exposure or errors were discarded. The stacked grayscale image was then thresholded manually, and a 3D isosurface was generated by a marching cubes algorithm in Nighres (Huntenburg et al., 2018). No image registration was performed, and the smoothness of the resulting image demonstrates the stability of the blockface imaging procedure. For the purpose of co-registration with the blockface stack, images were converted from color to a grayscale index of lightness and the contrast was rescaled so that intensities in the middle range of lightness (generally corresponding to gray matter regions) were brightest, while both brighter (white matter, background) and dark (artifact) regions became dark. The same approach was used on the blockface images. Grayscale images were then co-registered in 2D with the SyN algorithm from ANTs (Avants et al., 2008) using successively rigid, affine, and non-linear transformations, high levels of regularization as recommended for the subcortex (Ewert et al., 2019) and mutual information as cost function. After this transformation, intensity classification with FANTASM (Pham, 2001) was used to identify and background regions, and a second non-linear step of co-registration was applied. Because the tissue slice may have been flipped between cutting and staining, we repeated the co-registration with a flipped version of the stain images and kept the image with highest correlation with the blockface after alignment. Finally, the original stains lightness was transformed to match the blockface, and slice-by-slice intensity was linearly normalized. In the case of the Nissl stains, inhomogeneity correction with N4 (Tustison et al., 2010) was applied before intensity normalization. This did not influence border delineations. Processing was performed in python using the PIL, nibabel, and nighres libraries. We defined a tissue mask on both the blockface image and the stained sections using a fuzzy C-means algorithm and combining all classes with higher intensities. Both masks were then deformed and Dice factors (Dice, 1945) were calculated for the Nissl (0.88; standard error = 0.009; *n* = 128), and for Silver (0.92; standard error = 0.005; *n* = 129) stained sections. When co-registering microscopy slices to the corresponding blockface slice in 2D, the registration algorithm minimizes normalized mutual information. These normalizations provide a global linear scaling of the slice intensities, thus they do not change intensity relationships that would influence border delineations. While our registration procedure uses a state-of-the-art registration method based on mutual information and realistic deformation fields, it is limited. It provided visually acceptable results, but different stain contrasts or tissue damage may complicate the registration and require landmark-based manual alignment (Figure 8). Such problem can further be addressed using manual or automated segmentations of anatomical structures of interest. Checkerboard images for visual inspection of the registration quality are presented in Figure 9. For the 2D to 3D blockface, no registration is performed. 3D blockface to 3D post-mortem MRI registration is performed with normalized mutual information as well. Both 2D to 2D and 3D to 3D registration steps are performed with ANTs, a state-of-the-art registration algorithm for brain imaging (Klein et al., 2009; Avants et al., 2011).

## Discussion

We have developed a pipeline in which we processed an entire human brain combining UHF MRI with cryosectioning, followed by (immuno-)histological processing and subsequent 3D reconstructions. The pipeline differs from other valuable pipelines in its practical application. Most importantly, it is designed to minimize tissue deformation to facilitate 3D reconstructions through the application of MRI *in cranio* and the application of blockface imaging as an intermediate step in the reconstruction process. Additionally, the slicing of thick sections for histological and immunocytochemical processing allows for an approximate 10-fold reduction of the time that is needed to process a single specimen as compared to BigBrain (Amunts et al., 2013).

One advantage of the pipeline is that MRI is performed before brain extraction, preventing major tissue deformations at this stage, facilitating registration to available brain atlases. The incorporation of detailed blockface imaging allowed 3D reconstruction without registration which provides an important intermediate step facilitating co-registration of microscopy sections, which we performed as a final step. We did not measure the resulting section thickness during the cutting procedure. A study by Ito and Brill (1991) quantitatively assessed the variation in section thickness using autoradiography in large cryosections. They reported that the variation in thickness within sections was < 1 μm, and largely independent of the thickness of the cut sections. Since we cut 200 μm thick sections, the expected variation is < 0.5%. Additionally, Ito and Brill (1991) reported an interslice variation of 1.21 μm per section, which corresponds to 0.6% for 200 μm sections. Since the interslice variation is the result of both the imprecision of the downward feed of the knife and the intraslice variation, the interslice variation was mainly attributed to the unevenness of the cryosection surface. The staining procedure and coverslipping process are likely to have affected the thickness of the resulting embedded microscopy sections. Individual sections are registered to the blockface image using a 2D-2D registration. Potential differences in section thickness caused by the staining procedure were not reflected in the 3D reconstructions of the brain, since section thickness in the 3D reconstruction was determined by the blockface sections and not the stained microscopy slides. A number of challenges are associated with the study of human post mortem whole-head specimens using the current protocol. We obtained these tissues after tissue fixation. We therefore were unable to control the used approach, and therefore saline rinsing to remove blood coagulates was not performed. A major challenge is to avoid or remove air bubbles in the specimen by an appropriate preparation. We therefore tested the effect of trepanation on the midline of the skull, which only had limited positive effects. It is conceivable that air was introduced during the perfusion fixation, or after severing the head from the body. Air bubbles were still present after brain extraction, which could have provided to perform detailed scanning after autopsy. Additionally, we used OCT compound to embed the brain, which allowed an easy surface reconstruction based on the blockface images, although the results in the frontal pole showed some artifacts as a result of incomplete OCT coverage of the tissue.

The success of this approach is not only dependent on the quality of the tissue specimens, but also on the tailoring of the MR acquisition methods, since *post mortem* analyses of tissues require specific MR sequences and/or acquisition parameters (Sengupta et al., 2009; Miller et al., 2011; Weiss et al., 2015). The contrast resulting from fixed tissue differs from the appearance of fresh tissue on MRI, and it is important to note that changes occur in MRI relaxation times, including T1 and T2 shortening, as well as reduction of the magnetization transfer ratio, whereas the macromolecular proton fraction increases (Schmierer et al., 2008; Dawe et al., 2009; Birkl et al., 2016). In our previous studies, we developed a multi-stage tissue scanning protocol which allows the spatial normalization of small tissue blocks into Montreal Neurological Institute (MNI-) space (Weiss et al., 2015). The imaging of smaller tissue blocks at a high level of detail is valuable, but registration of the anatomical results to MNI-standard space and/or the whole brain is a challenge as a result of the limited shared information between the tissue blocks and the MNI-template. The application of a multistage approach, in which tissue was carefully imaged, cut and then each stage was imaged again individually, facilitated registration of the tissue into MNI-space (Weiss et al., 2015). These efforts have resulted in a pipeline, in which we can quantitatively compare *post mortem* MRI results with MRI validation experiments using microscopy approaches (Forstmann et al., 2017). We now present the next step, and have extended our pipeline to allow UHF scanning of larger specimens, with subsequent tissue processing for histology and immunocytochemistry, as well as 3D reconstructions of the data. The pipeline is developed in a way that it can easily be adapted to accommodate specific requirements for other research groups, and can be used for studies in both non-demented control as well as diseased tissue. For example, the tissue processing part of the protocol can be combined with MRI imaging using lower field strengths (e.g., 3T), in which the moderate SNR penalty can be addressed using longer scan times. Additionally, the protocol can be scaled up by including more specimens, since the cutting of the tissue was done in 3 days, resulting in approximately 850 individual 200 μm thick sections. Full reconstruction of a series of human brains, requiring the staining of each individual section becomes more feasible with 200 μm sections, reducing the amount of work 10x as compared to the approach described by Amunts et al. in which 7404 consecutive 20 μm paraffin sections were cut to create BigBrain (Amunts et al., 2013).

A number of alternative approaches to study the detailed anatomy of the human brain are available: More classical invasive investigations of tissue specimens including microscopy approaches require tissue processing, which leads to the loss of information on the 3D structure of the specimen. For MRI in addition to scanning of small, formalin-fixed brain tissue blocks, increasing numbers of studies investigating whole brain specimens extracted from the skull and scanning for an extended period of time (hours or days) are being published (Larsson et al., 2004; Pfefferbaum et al., 2004; McNab et al., 2009; Miller et al., 2011; Annese et al., 2014). Other protocols have also shown the benefit of the use of thicker sections to increase the tissue processing efficiency (Alegro et al., 2016; Alho et al., 2017, 2018), and providing improved detection of brain nuclei as compared to MRI results. Published methods each have their merits, and at the same time have substantial differences in MRI acquisition parameters, tissue processing steps, registration methods, and individual brain donors, which complicates an in depth comparison between methods. Additionally, we feel that it is important to note that upscaling of the experimental approach is associated with an increase in financial costs. Additionally, non-invasive autopsy is becoming an increasingly popular alternative to invasive autopsy procedures (Roberts et al., 2012; Bolliger and Thali, 2015). *In situ* human brain scans can be acquired without tissue fixation after the demise of a subject. These scans are usually short, since the available time is limited due to the commencing decay of the tissue (Dashner et al., 2003; Tenenholz Grinberg et al., 2009). We would like to note that in our tissues we have not acquired MRI images before tissue fixation, and we therefore cannot compare MRI quality before and after fixation. The use of fixed tissue prevents unwanted long post mortem intervals, which negatively affect histological and immunohistochemical quality. Additionally, the use of fixed tissue has a number of practical advantages including scheduling of scan time, and availability of archival donor material as compared to prospective collection of fresh material. Advantages of *in vivo* MRI include the possibility to follow development across the lifespan, availability of participants for these studies, and the possibilities to compare between groups of interest. Limitations of *in vivo* approaches include movement artifacts and limited scan duration, which substantially reduces the anatomical detail that can be obtained (Callaghan et al., 2015). To date, detailed *in vivo* anatomical scans cannot always provide the anatomical detail needed to investigate small deep brain structures. The development of novel *post mortem* approaches therefore will continue to provide an invaluable addition to the field of human neuroscience.

## Data Availability Statement

Blockface datasets are available via: 10.21942/uva.12496853.v1.

## Ethics Statement

The studies involving human participants were reviewed and approved by all the donors who provided written informed consent for whole body donation prior to death. The tissue was obtained in accordance with the Dutch Burial and Cremation act. Additional approval for these studies was obtained from the Ethical Board of the Medical Faculty of the University of Leipzig (153/17-ek).

## Author Contributions

AA, RB, RLAWB, and AH were responsible for tissue processing. KP, EK, P-LB, MK, MM, JG, RT, NW, HM, and BF contributed to the MRI scanning. P-LB created 3D reconstructions. AA and BF wrote the manuscript. All authors edited the manuscript.

## Conflict of Interest

The authors declare that the research was conducted in the absence of any commercial or financial relationships that could be construed as a potential conflict of interest.

## Footnotes

1 http://www.fil.ion.ucl.ac.uk/spm/

2 http://hmri.info
